# Removal of a Shot from the Eye Six Months after Injury

**Published:** 1888-02

**Authors:** Wm. Caston

**Affiliations:** Corsicana, Texas


### REMOVAL OF A SHOT FROM THE EYE SIX MONTHS AFTER ENTRY.

*By Wm. Caston, M. D., Corsicana, Texas.*

For Daniel's Texas Medical Journal.

DURING last February Mr. M. while out with several friends hunting birds, in the act of shooting with gun to right shoulder and left eye closed, a glancing shot from the gun of one of the party struck the nasal side of the cornea of his open right eye, causing great pain and immediate loss of vision in that eye. The physician who saw him a few hours later, described the cornea as presenting all the appearance of the shot having entered and passed through the eye from the inner or nasal side of the cornea to the outer or temporal side of the ciliary region, the point of entrance being jagged, and protruded therefrom a white viscid substance. Not seeing the case at that time, I can form a very poor conception of its appearance. Seven weeks after the accident I obtained the forgoing history from the patient, and carefully examining the eye, finding considerable sub-conjunctival injection at the outer ciliary margin, but limited to a small space, the cornea comparatively clear, showing only a haziness where the shot was said to have struck, the iris dull and lustreless and pupil narrowed and sluggish, in fact all the ordinary appearance of iritis, which had existed since the receipt of the injury. Through the contracted pupil could be plainly seen an opaque lens. What the treatment had been, I did not learn or have forgotten, except that a patch had been worn over the eye constantly and some drops instilled into it, principally cocaine, with possibly, atropine, but evidently not sufficient to over come the contractions of the pupil. I decided the shot had not entered the eye, but glanced and so wounded the point of present redness as to set u, iritis and produce traumatic cataract, and advised the treatment accordingly.

I saw no more of the case, he remaining under the treatment of the physician who first saw him till August 15, nearly five months later, when he again presented himself at my office, and said the shot was in his eye and could now be seen, which I found to be true on examination. Rolling freely around in the clear aqueous of the anterior chamber as bright and smooth and almost as spherical as new, not seeming to have caused the least irritation, was the object of all this man's suffering. He said he had continued the treatment he was under when last at my office, adding blue glasses as I advised at the time, in place of the patch; had suffered a great deal and used much cocaine in the eye, and morphine internally, up to within two months of the present time, when all inflammatory symptoms seemed to subsides and the eye had given him no special annoyance since; though he could not see with it, till several weeks past, when ne began to perceive light, and now could discover large objects. The shot had rolled forward through the pupil without his knowledge and he had no intimation of it before his attention was called to it by a bystander, though evidently it had not occupied its present position long. as subsequent events would indicate. There was as I have said, apparently not the slightest irritation, the aqueous being perfectly clear, the iris bright and no conjunctival disturbance, but the point of entry of the shot was now plainly marked by a dense leucoma, and under oblique illumination, secondary cataract of the posterior capsule of lens was evident, which probably accounted for impairment of vision. I advised an operation for immediate removal of the shot. He had been advised by his physician "to wait its gravitation," "when," he said "it would be easier to remove, and the incision which would leave a permanent scar would not have to be made over the sight," and my advice was not accepted.

The following day he was at my office early, complaining of pain and uneasiness in the eye. The shot had "gravitated," and lay buried in the iris at the most dependent point of the anterior chamber, being dimly seen through the now cloudy aqueous; the iris was dull, and cornea surrounded by the characteristic zone of injected blood vessels. Iritis was fully developed. After fortifying myself with a doubtful prognosis as to the results from an operation, at the same time assuring the patient it was the only alternative, at his request I proceeded under cocaine to remove the shot by introducing a kerotome at the lower schlero-corneal junction so as the edge of the incision would just reach the shot, hoping to be able to grasp and withdraw it with a delicate pair of forceps, knowing it would no longer roll out with the escape of aqueous as I was satisfied it would have done the day previous before inflammation set up. After several vain attempts at extraction with forceps, having as often enlarged the first incision with scissors, the shot passed through the iris in which it had imbeded itself, and disappeared in the posterior chamber; the pressure of the non-collapsed cornea assisting my forceps to force it through. Placing the patient in the upright position, I could see the shot under oblique illumination as it rolled to the most dependent point. Introducing a David's spoon and passing it under, a No. 7 shot rolled out and dropped on the floor.

After closing an extensive iridectomy, removing all the mutilated iris, I applied ice cloths. I could not hope to escape now a very serious reaction, inflammation being already developed; but with alternate hot and cold applications cocaine, and atropia in the eye and during the paroxysms of pain, leeches to the temple with hypodermic injections of morphine as necessary, in three weeks he was again able to attend my office, but has never been able to see with the eye, the pupil being now occluded, as well as the calaboma of iris made in the iridectomy by inflammatory products.

I was not aware before this case came under my observation, that a foreign body could remain so long—not encapsulated—in the eye, without completely destroying it, and I have reason to believe in this instance that the body was not encapsulated. It remained undiscovered for more than six months, and after subsidence of the inflammation immediately following the injury, the patient himself was not cognizant of its presence, experiencing no unusual sensation from its rolling around in the lens chamber where it had undoubtedly been since its entrance, causing gradual disintegration solution and absorption of the lens matter, and where it would probably have never caused further trouble, a tolerance for its presence being formed, had it remained, and after passing forward through the pupil into the anterior chamber, it created no disturbance until it had lodged itself and been pressed by the cornea into the substance of the iris. Cases are recorded where foreign bodies have remained a much longer time—encapsulated—in the anterior chamber without exciting serious injury. Saemish records one where he removed a fragment of stone from the anterior chamber twelve years after its entering. It had originally been lodged in the lens as the shot in the present instance, and after absorption of the lens, fell forward into the anterior chamber, but remained attached to the lens capsule by a filament of the tissue encapsulating it.

DeWecker also notes a similar extraction after fourteen years stay in the same position before exciting injury, but in these cases the body was encapsulated, and in this as I have said, I do not believe it was. I report this case thus in detail, because I have seen no exactly parallel one recorded.

January 12, 1888.

